# 基于金属有机骨架多孔碳材料的分散固相萃取-超高效液相色谱-串联质谱法测定水中4种酚类内分泌干扰物

**DOI:** 10.3724/SP.J.1123.2023.05012

**Published:** 2024-03-08

**Authors:** Pan WANG, Jiping MA, Shuang LI, Jiawen CHENG, Chaonan HUANG

**Affiliations:** 青岛理工大学环境与市政工程学院, 山东 青岛 266525; School of Environmental and Municipal Engineering, Qingdao University of Technology, Qingdao 266525, China

**Keywords:** 超高效液相色谱-串联质谱, 金属有机骨架材料, 分散固相萃取, 酚类内分泌干扰物, 环境水体, ultra performance liquid chromatography-tandem mass spectrometry (UPLC-MS/MS), metal-organic framework materials, dispersive solid-phase extraction (DSPE), phenolic endocrine-disrupting chemicals, environmental waters

## Abstract

酚类内分泌干扰物是一种干扰内分泌系统的外源性物质,其进入生物体后会干扰细胞的正常功能,引起生殖发育毒性,因此亟需开发一种快速、灵敏的分析方法,用于环境水体中酚类内分泌干扰物的检测。本研究采用溶剂热法合成了一种由金属有机骨架衍生的多孔碳材料(UiO-66-C),并将其作为萃取吸附剂来富集水中的4种酚类内分泌干扰物(双酚A、4-叔辛基苯酚、4-壬基酚、壬基酚)。建立了一种分散固相萃取(DSPE)结合超高效液相色谱-串联质谱(UPLC-MS/MS)测定水中酚类内分泌干扰物的分析方法。通过扫描电子显微镜、X射线衍射及傅里叶变换红外光谱等测试方法对UiO-66-C进行表征,证明了该材料的成功制备。对DSPE条件进行优化,包括UiO-66-C用量、水样pH、吸附时间、洗脱液种类及体积、洗脱时间和离子强度。在最佳实验条件下,4种酚类内分泌干扰物在0.5~100 μg/L范围内线性关系良好,方法检出限和定量限分别为0.01~0.13 μg/L和0.03~0.42 μg/L,日内和日间精密度分别为1.5%~10.6%和6.1%~13.2%。将该方法应用于自来水和地表水的检测,4种酚类内分泌干扰物的加标回收率为77.1%~116.6%;在自来水样品中未检测到4种酚类内分泌干扰物,而在地表水中检测到微量的4-壬基酚和壬基酚,检出水平分别为1.38 μg/L和0.26 μg/L。该方法具有良好的准确度和精密度,为环境水体中酚类内分泌干扰物的检测提供了一种快速、高效、灵敏的新途径。

## Abstract (en)

Phenolic endocrine-disrupting chemicals (EDCs) are exogenous substances that interfere with the endocrine system and disrupt normal cell functions upon entering a living organism, leading to reproductive and developmental toxicity. Therefore, the development of a rapid and efficient analytical method for detecting phenolic EDCs in environmental waters is crucial. Owing to the low concentration of phenolic EDCs in environmental water, appropriate sample pretreatment methods are necessary to remove interferences caused by the sample matrix and enrich the target analytes before instrumental analysis. Dispersive solid-phase extraction (DSPE) has gained considerable attention as a simple and rapid sample pretreatment method for environmental-sample analysis. In this method, an adsorbent material is uniformly dispersed in a sample solution and the target analytes are extracted through processes such as vortexing. Compared with traditional solid-phase extraction (SPE), DSPE increases the contact area between the adsorbent and sample solution, reduces the required amounts of adsorbent and organic solvents, and improves the extraction efficiency. The adsorbent material plays a critical role in DSPE because it determines the extraction efficiency of the method. Metal-organic frameworks (MOFs) are porous framework materials composed of metal clusters and multifunctional organic ligands. They possess many excellent properties such as tunable pore sizes, large surface areas, and good thermal and chemical stability, rendering them ideal adsorbent materials for sample pretreatment. MOF-derived porous carbon materials obtained through high-temperature carbonization not only increase the density of MOF materials for better separation but also retain the advantages of a large surface area, highly ordered porous structure, and high porosity. In this study, a porous carbon material derived from an MOF, named as University of Oslo-66-carbon (UiO-66-C), was synthesized using a solvothermal method and applied as an adsorbent to enrich four phenolic EDCs (bisphenol A, 4-*tert*-octylphenol, 4-nonylphenol, and nonylphenol) in water. A method combining DSPE with ultra performance liquid chromatography-tandem mass spectrometry (UPLC-MS/MS) was established to analyze these phenolic EDCs in water. The UiO-66-C dosage, pH of water sample, adsorption time, eluent type and volume, elution time, and ion strength were optimized. Gradient elution was performed using methanol-water as the mobile phase. The target analytes were separated on an ACQUITY UPLC BEH C18 column (100 mm×2.1 mm, 1.7 μm), and multiple reaction monitoring (MRM) was conducted in negative electrospray ionization mode. The method exhibited a linear correlation within the range of 0.5-100 μg/L for the four phenolic EDCs. The limits of detection (LODs) and quantification (LOQs) of the four phenolic EDCs were 0.01-0.13 μg/L and 0.03-0.42 μg/L, respectively. The precision of the method was evaluated through intra- and inter-day relative standard deviations (RSDs), with values ranging from 1.5% to 10.6% and from 6.1% to 13.2%, respectively. When applied to the detection of phenolic EDCs in tap and surface water, the spiked recoveries of the four phenolic EDCs were 77.1%-116.6%. Trace levels of 4-nonylphenol and nonylphenol were detected in surface water at levels of 1.38 and 0.26 μg/L, respectively. The proposed method exhibits good accuracy and precision; thus, it provides a new rapid, efficient, and sensitive approach for the detection of phenolic EDCs in environmental water.

近年来,内分泌干扰物(endocrine-disrupting chemicals, EDCs)受到了广泛关注,这些化学物质可以模拟或干扰动物体内内源性激素的作用,与雌激素受体结合或抑制正常的生物反应,即使在微量的情况下也会破坏生物体的正常功能,导致生长发育受损^[1,2]^。双酚A(BPA)、4-叔辛基苯酚(4-*t*-OP)、壬基酚(NP)和4-壬基酚(4-NP)是4种典型的酚类EDCs;其中,BPA主要用于制造聚碳酸酯塑料和环氧树脂,常用在食品接触材料中(如饮料瓶和婴儿奶瓶); 4-*t*-OP、NP和4-NP在工业产品(如衬垫漆、黏合剂、塑料、饮料包装和食品罐)制造中有着广泛的应用^[3⇓-5]^, 4种酚类EDCs的结构与性质如表1所示。酚类EDCs具有高疏水性和难降解性,其进入污水处理厂后难以降解,被直接排放至环境水体中,从而导致其在水生生态系统中被频繁检出。《中国生活饮用水卫生标准》(GB 5749-2022)^[6]^规定BPA的含量限值为10 μg/L,美国环境保护署(EPA)地表水水质标准规定NP的含量限值为6.6 μg/L^[7]^。因此,建立一种快速、高效、灵敏的分析方法来测定环境水体中酚类EDCs的含量非常必要。目前,气相色谱-质谱法(GC-MS)^[8]^、高效液相色谱法(HPLC)^[9]^和超高效液相色谱-串联质谱法(UPLC-MS/MS)^[10,11]^是测定酚类EDCs的主要分析方法;其中,与GC-MS和HPLC相比, UPLC-MS/MS的灵敏度更高,更适合水中痕量污染物的分析检测。

**表 1:** 4种酚类EDCs的结构与性质

Analyte	Structure	Molecular mass/(g/mol)	pK_a_	log K_ow_
Bisphenol A (BPA)	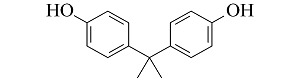	228.29	9.73	3.43
4-tert-Octylphenol (4-t-OP)	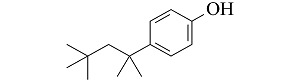	206.32	10.20	4.93
4-Nonylphenol (4-NP)	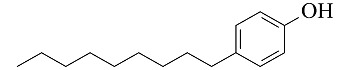	220.35	10.30	5.76
Nonylphenol (NP)	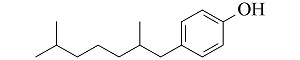	220.35	10.25	5.71

p*K*_a_: acidity coefficient; *K*_ow_: octanol-water partition coefficient.

在进行色谱分析之前,需要进行样品前处理步骤,以便从样品基质中有效地提取目标分析物并提高分析灵敏度。目前,水中酚类EDCs的样品前处理方法主要有固相萃取(SPE)^[12⇓-14]^、固相微萃取(SPME)^[15]^、搅拌棒吸附萃取(SBSE)^[16]^和磁固相萃取(MSPE)^[7,17]^等。除此之外,分散固相萃取(DSPE)在环境样品的分析中得到了广泛关注,DSPE首先将吸附剂材料均匀地分散在样品溶液中,再通过涡旋等方式萃取目标分析物,是一种简单、快速的样品前处理方法。与传统的SPE相比,DSPE增大了吸附剂与样品溶液的接触面积,萃取吸附剂和有机溶剂的用量更少,萃取效率更高。在DSPE过程中,吸附材料起着关键作用,它决定了目标分析物的萃取效率。

金属有机骨架材料(MOFs)是由金属中心和有机配体自组装形成的一类新型多孔晶体材料,具有比表面积大、孔隙率高、结构可调节和稳定性好等特性,是一类理想的吸附材料^[18]^。我们课题组^[19⇓⇓-22]^制备了MOF-5、UiO-66-NH_2_、MIL-53、MIL-101等多种MOFs复合材料,用于环境水样中除草剂、农药等污染物的富集分析。由于MOFs的密度相对较小,在样品前处理过程中难以实现固液分离,而经高温碳化得到的MOFs多孔碳材料不仅能够增大MOFs材料的密度,使其利于分离,还具有更高的化学稳定性和力学强度^[23,24]^。此外,MOFs多孔碳材料保留了MOFs高比表面积、高孔隙率的性质以及高度有序的多孔结构,一些MOFs(如MOF-5^[25,26]^、ZIF-8^[27,28]^、MIL-53^[29]^、MOF-235^[30]^、ZIF-67^[31]^等)已被成功用于制备纳米多孔碳材料,并在气体储存、催化、传感以及生物大分子的纯化富集等方面表现出较好的应用潜力。

UiO-66是一种具有多孔网络结构的MOF材料^[32]^,由金属中心锆和配体对苯二甲酸自组装而成。UiO-66具有良好的水热稳定性、可调控的孔径和高比表面积,是合成纳米多孔碳的一种理想前驱体^[33]^。本研究采用溶剂热法合成了UiO-66,然后在高温下碳化得到MOF多孔碳材料(UiO-66-C),利用扫描电子显微镜(SEM)、X射线衍射(XRD)和傅里叶变换红外光谱(FT-IR)等测试手段对其进行表征,并优化了影响DSPE效果的主要因素。将涡旋辅助DSPE与UPLC-MS/MS相结合,建立了一种快速、灵敏富集分析水中酚类EDCs的方法。

## 1 实验部分

### 1.1 仪器、试剂与材料

QTRAP 3500超高效液相色谱-三重四极杆质谱仪(美国AB Sciex公司); QSL-1800X-S真空管式炉(合肥科晶材料技术有限公司); XW-18D+漩涡混合器(绍兴市苏珀仪器有限公司); Millipore D-24UV超纯水机(美国Millipore公司); DZF-6020真空干燥箱(上海精宏实验设备有限公司); Sigma 300扫描电子显微镜(德国Zeiss公司); SmartLab9K X射线衍射仪(日本Rigaku公司); Frontier傅里叶变换红外光谱仪(美国PerkinElmer公司); 3H2000-PS2表面积及孔径分析仪(北京贝士德仪器科技有限公司); ZetaPlus电位仪(美国Brookhaven公司)。

酚类EDCs标准品:BPA(纯度≥99.8%)购自北京百灵威科技有限公司,4-*t*-OP(纯度≥98%)购自北京沃凯生物科技有限公司,NP(纯度≥99%)购自上海阿拉丁生化科技有限公司,4-NP(纯度≥99%)购自德国Dr. Ehrenstorfer有限公司。乙醇(分析纯)、盐酸(分析纯)、氯化锆(纯度99.5%)和对苯二甲酸(纯度99%)购自上海麦克林生化科技股份有限公司,甲醇(色谱纯)购自德国默克公司,二氯甲烷(色谱纯)购自天津市科密欧化学试剂有限公司,甲醇(分析纯)和*N*,*N*-二甲基甲酰胺(DMF)购自天津市富宇精细化工有限公司。

自来水和地表水采集于青岛某地,两种水样经0.45 μm滤膜过滤后,在4 ℃下保存于棕色玻璃瓶中。

### 1.2 标准溶液的配制

称取4种酚类EDCs标准品各10 mg(精确至0.1 mg),用甲醇溶解并定容至10 mL,分别配制成质量浓度为1000 mg/L的标准储备液,置于棕色玻璃小瓶中,于4 ℃下冷藏保存。移取各标准储备液1 mL,用甲醇稀释并定容至10 mL,得到质量浓度为100 mg/L的4种酚类EDCs混合标准工作液,于4 ℃下冷藏保存。

### 1.3 UiO-66-C的制备

UiO-66-C的制备分为以下两个步骤:(1)采用溶剂热法制备UiO-66^[34]^,首先将1.166 g氯化锆和0.8307 g对苯二甲酸加入至60 mL DMF中,经超声溶解后将上述溶液转移至反应釜内衬中,在120 ℃下反应24 h;之后将上述反应体系冷却至室温,用DMF和乙醇将所得材料进行洗涤并离心收集,在70 ℃下真空干燥得到白色粉末状的UiO-66;(2)将制备好的UiO-66粉末装于瓷舟中,置于管式炉的中段位置,通入氮气并以5 ℃/min的速率升温至800 ℃,保持3 h;待管式炉冷却至室温后将瓷舟取出,所得到的黑色粉末即为UiO-66-C。

### 1.4 样品前处理

将15 mg UiO-66-C置于离心管中,加入50 mL含有目标化合物的水样,再加入1.0 g NaCl(最终质量分数为2%),并使用盐酸调节水样pH至5,涡旋处理4 min,在4000 r/min下离心5 min,弃去上清液,然后加入1.0 mL甲醇-二氯甲烷(3:7, v/v)洗脱液,涡旋洗脱1.5 min,收集洗脱液并通过0.22 μm滤膜过滤,之后进行UPLC-MS/MS分析,样品前处理流程图见图1。

**图 1:**
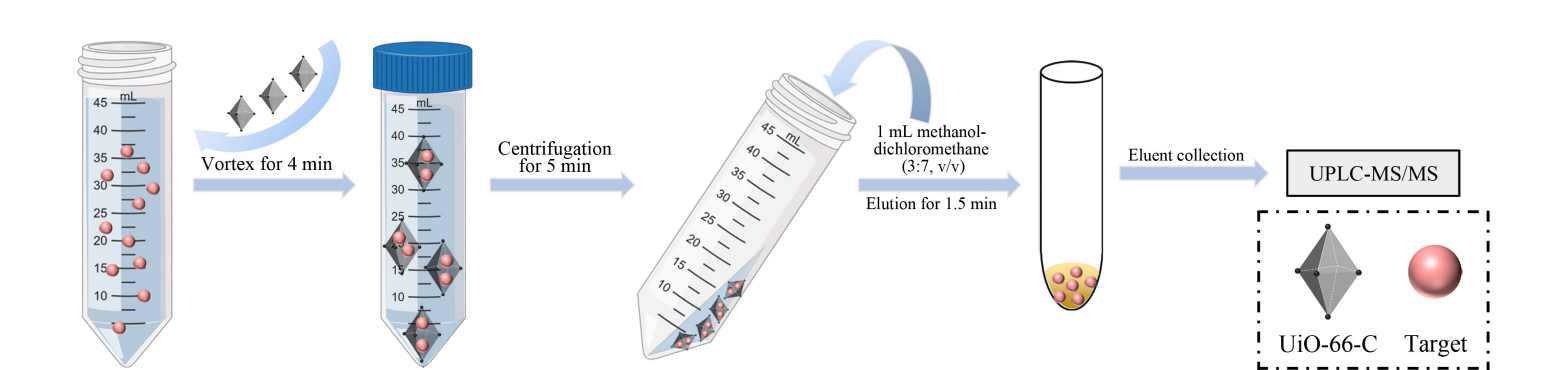
样品前处理流程图

### 1.5 仪器条件

ACQUITY UPLC BEH C18色谱柱(100 mm×2.1 mm, 1.7 μm;美国Waters公司);柱温40 ℃;进样量5 μL;流动相:A相为水,B相为甲醇,流速0.3 mL/min。梯度洗脱程序:0~2.0 min, 80%A; 2.0~5.0 min, 80%A~10%A; 5.0~8.0 min, 10%A; 8.0~8.1 min, 10%A~80%A; 8.1~10.0 min, 80%A。

电喷雾电离(ESI)源,负离子模式;数据采集模式:多反应监测(MRM);离子源温度:500 ℃;离子源电压:-4 kV;气帘气压力:2.07×10^5^ Pa;雾化气压力:3.45×10^5^ Pa;辅助气压力:4.14×10^5^ Pa。4种酚类EDCs的其他质谱参数见表2。

**表 2:** 4种酚类EDCs的质谱参数

Analyte	t_R_/ min	Precursor ion (m/z)	Product ions (m/z)	DPs/ V	CEs/ eV
BPA	5.85	226.9	212.0^*^, 132.9	-82, -87	-27, -41
4-t-OP	6.85	205.1	132.8^*^, 147.1	-101, -101	-27, -27
4-NP	7.29	219.0	105.7^*^, 118.0	-90, -103	-27, -26
NP	7.95	219.0	133.0^*^, 146.9	-83, -89	-41, -37

* Quantitative ion; DP: declustering potential; CE: collision energy.

## 2 结果与讨论

### 2.1 材料表征

利用SEM观察UiO-66和UiO-66-C的形貌,结果如图2所示,UiO-66和UiO-66-C均呈现纳米立方体结构,说明碳化过程不会破坏UiO-66的结构^[34]^。

**图 2:**
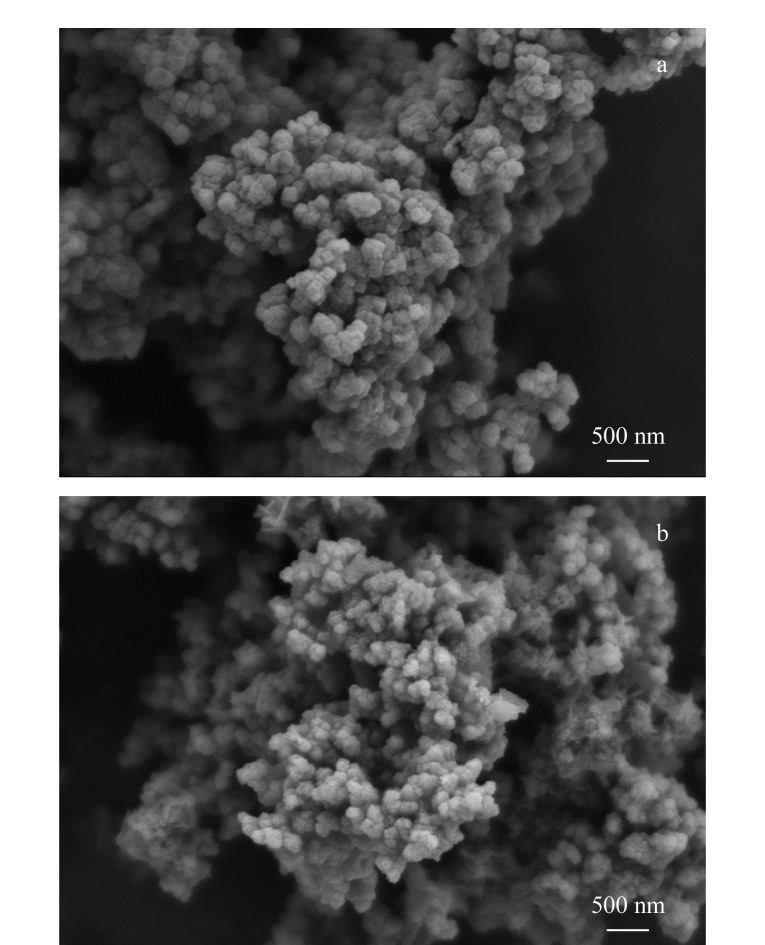
(a)UiO-66和(b)UiO-66-C的SEM照片

利用FT-IR对UiO-66和UiO-66-C进行表征,结果如图3a所示。在UiO-66中,处于660、747和1018 cm^-1^位置的特征峰归因于Zr-O键的振动,1656 cm^-1^处的特征峰归因于对苯二甲酸中C=O键的伸缩振动;此外,1507 cm^-1^和1576 cm^-1^处的特征峰是由苯环上C=C的伸缩振动引起的。在UiO-66-C中,1620 cm^-1^处的振动峰归因于C=C键的伸缩振动;在494 cm^-1^附近可观察到一个明显的吸收峰,归因于O-Zr-O键的振动,表明UiO-66-C中可能存在大量的ZrO_2_,这是因为在碳化过程中,UiO-66中的金属中心转化成了ZrO_2_^[35]^。以上FT-IR结果证实了UiO-66和UiO-66-C的成功制备,且与文献[34]报道的结果一致。

**图 3:**
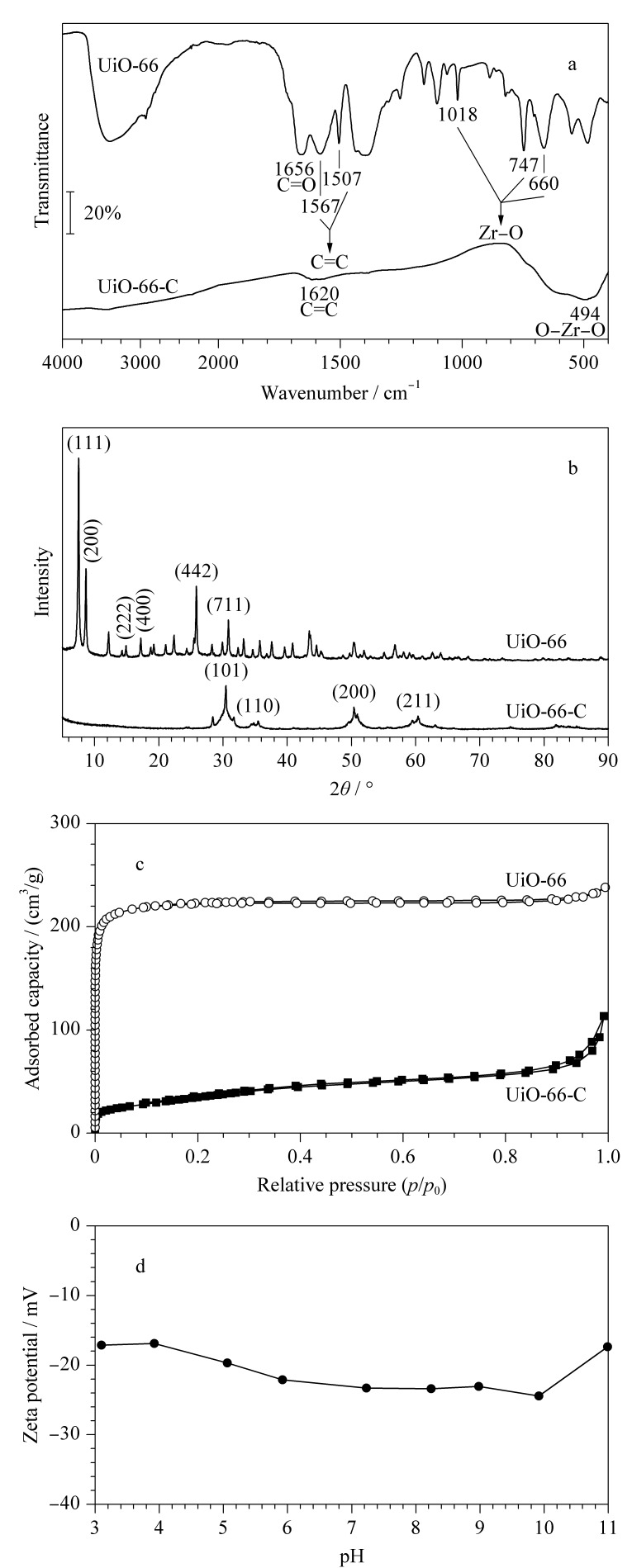
UiO-66和UiO-66-C的(a)FT-IR图、(b)XRD图、(c) N_2_吸附-脱附等温曲线和(d) UiO-66-C的Zeta电位图

利用XRD对UiO-66和UiO-66-C的晶体结构进行表征,结果如图3b所示。UiO-66的结构中存在(111)、(200)、(222)、(400)、(442)和(711)6个晶面;在经过碳化处理之后,UiO-66中的尖锐衍射峰不再出现,相反在UiO-66-C的结构中观察到4个新的衍射峰,分别对应于(101)、(110)、(200)和(211)4个晶面,表明UiO-66-C属于四边形的晶体结构。以上结果与之前的文献[34]报道一致,证实了UiO-66和UiO-66-C的成功制备。

采用N_2_吸附-脱附等温曲线研究了碳化处理对UiO-66比表面积、孔隙体积和孔径的影响,结果如图3c和表3所示。在UiO-66-C的N_2_吸附-脱附等温曲线中,在饱和蒸汽压力附近出现轻微的滞后环,表明UiO-66-C的结构中存在介孔。相比于UiO-66, UiO-66-C的比表面积和孔隙体积均明显下降,分别为127.87 m^2^/g和0.17 cm^3^/g,而UiO-66-C的平均孔径从2.18 nm增加到5.26 nm,这可能是由于碳化过程中UiO-66中的有机配体经热分解后生成了CO_2_等气体,导致UiO-66的骨架坍塌^[36]^,扩大了孔径。UiO-66-C结构中的孔径有利于目标物在吸附材料中扩散,从而提高萃取效率。此外,实验对UiO-66-C的Zeta电位也进行了测定,结果如图3d所示,UiO-66-C在pH为3~11的水溶液中呈电负性。

**表 3:** UiO-66和UiO-66-C的比表面积、孔隙体积及孔径

Material	Specific surface area/(m^2^/g)	Pore volume/ (cm^3^/g)	Pore size/ nm
UiO-66	671.64	0.36	2.18
UiO-66-C	127.87	0.17	5.26

### 2.2 前处理条件的优化

为了获得最佳萃取效果,实验对影响萃取回收率的各种因素进行了考察与优化,主要包括UiO-66-C用量、水样pH、吸附时间、洗脱液种类及体积、洗脱时间和离子强度等。在每个优化条件下进行3次独立的平行实验,水样中酚类EDCs的加标水平为40 μg/L。

#### 2.2.1 UiO-66-C用量

考察不同质量(5、10、15、20、25 mg)的UiO-66-C吸附剂对4种酚类EDCs萃取回收率的影响。结果如图4a所示,随着UiO-66-C的质量从5 mg增加至15 mg, 4种酚类EDCs的萃取回收率逐渐增大;然而,当UiO-66-C的质量从15 mg增加至25 mg时,4种酚类EDCs的萃取回收率基本保持平稳,表明UiO-66-C对酚类EDCs的吸附已达到饱和。因此,确定最佳UiO-66-C用量为15 mg。

#### 2.2.2 水样pH

样品溶液的pH是影响酚类EDCs萃取回收率的一个关键因素,它可以改变目标物的现有形态,并影响吸附剂的稳定性。在不同的pH(3、5、7、9、11)条件下考察了水样pH对酚类EDCs萃取回收率的影响。如图4b所示,水样pH值从3增加至5, 4种酚类EDCs的萃取回收率均有所提高;当水样pH为5~9, 4种酚类EDCs的萃取回收率略有下降;随着水样pH增加至11, 4种酚类EDCs的萃取回收率均明显下降。当水样pH低于4种酚类EDCs的酸度系数(p*K*_a_)时,4种酚类EDCs主要以分子形态存在于水样中;当水样pH大于4种酚类EDCs的p*K*_a_时,BPA、4-*t*-OP、4-NP、NP均失去一个质子,以负离子形态存在于水样中^[37]^。由UiO-66-C的Zeta电位结果可知,在pH为3~11时,UiO-66-C带负电;但当水样pH>10时,UiO-66-C与4种酚类EDCs之间会存在静电排斥作用,导致萃取回收率下降。因此,最终确定水样的最佳pH为5。

#### 2.2.3 吸附时间

吸附时间是影响萃取回收率的重要因素。实验考察了不同吸附时间(1、2、3、4、5 min)对4种酚类EDCs萃取回收率的影响。结果如图4c所示,随着吸附时间的延长,4种酚类EDCs的萃取回收率逐渐升高;在吸附时间达到4 min后,进一步延长吸附时间,4种酚类EDCs的萃取回收率没有明显提高。因此,选择4 min为最佳吸附时间。

**图 4:**
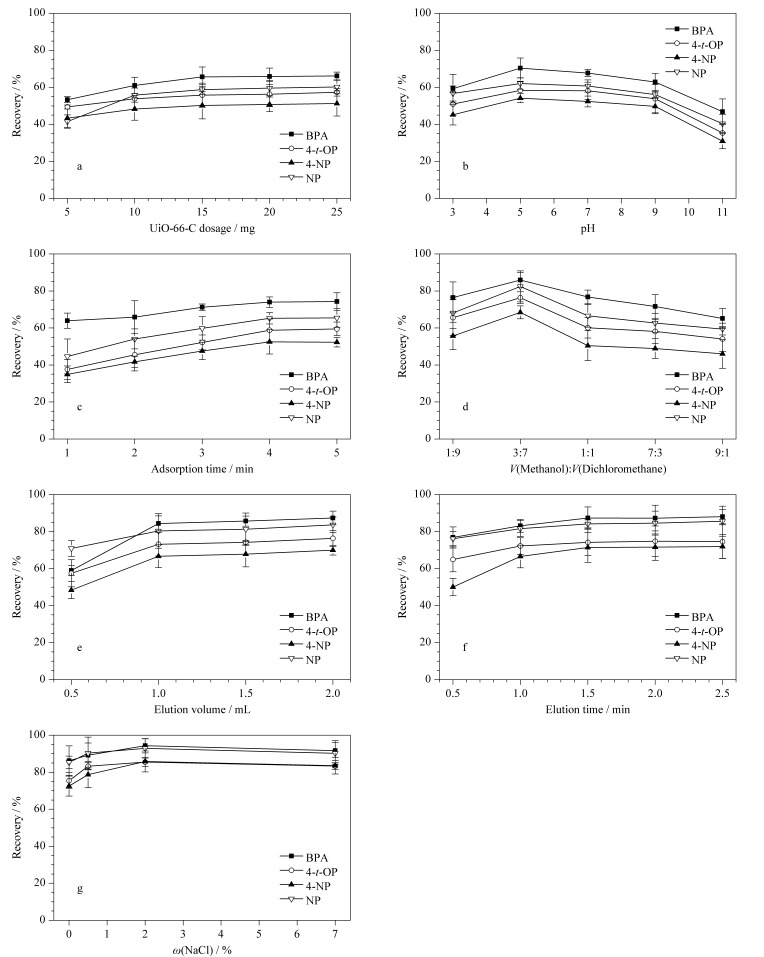
(a)UiO-66-C用量、(b)水样pH、(c)吸附时间、(d)洗脱液、(e)洗脱液体积、(f)洗脱时间和(g)NaCl质量分数对4种酚类EDCs回收率的影响(*n*=3)

#### 2.2.4 洗脱液种类

不同类型的洗脱液会对目标物的回收率产生影响。实验中分别使用甲醇、乙腈、丙酮、乙酸乙酯、二氯甲烷及甲醇-二氯甲烷(1:1, v/v)等有机溶剂对4种酚类EDCs进行洗脱。实验结果表明,与其他溶剂相比,甲醇-二氯甲烷(1:1, v/v)的洗脱效果最好,这可能是因为BPA、4-*t*-OP、4-NP和NP可以与甲醇形成氢键作用力,且上述化合物均具有一定的疏水性(log *K*_ow_>3),而二氯甲烷对疏水性化合物的溶解度较好,通过混合两种溶剂可以使样品中的化合物得到更好的溶解。因此,选择甲醇-二氯甲烷(1:1, v/v)进行后续比例优化。实验评估了不同体积比(1:9、 3:7、 1:1、 7:3、 9:1)的甲醇-二氯甲烷对4种酚类EDCs回收率的影响,结果如图4d所示。当使用甲醇-二氯甲烷(3:7, v/v)时,4种酚类EDCs的回收率最高,因此确定甲醇与二氯甲烷的体积比为3:7。

#### 2.2.5 洗脱液体积

为了进一步提高洗脱效率,考察了甲醇-二氯甲烷(3:7, v/v)体积对4种酚类EDCs回收率的影响。分别使用4种不同体积(0.5、1.0、1.5、2.0 mL)的洗脱液进行洗脱,结果如图4e所示,当洗脱液体积从0.5 mL增加至1.0 mL时,4种酚类EDCs的回收率明显提高;当洗脱液体积继续增加至2.0 mL时,4种酚类EDCs的回收率趋于平稳,表明1.0 mL洗脱液足以洗脱目标物。因此,确定洗脱液的体积为1.0 mL。

#### 2.2.6 洗脱时间

实验还评估了不同洗脱时间(0.5、1.0、1.5、2.0、2.5 min)对4种酚类EDCs回收率的影响。结果如图4f所示,当洗脱时间从0.5 min增加至1.5 min时,4种酚类EDCs的回收率得到大幅提升;然而,当洗脱时间继续延长至2.5 min时,4种酚类EDCs的回收率没有明显变化。因此,最终确定最佳洗脱时间为1.5 min。

#### 2.2.7 离子强度

盐的加入可以改变溶液的离子强度、渗透压和黏度,从而影响目标化合物在水样中的扩散速率。实验考察了水样中不同质量分数(0、0.5%、2%和7%)的NaCl对4种酚类EDCs回收率的影响。由图4g可知,随着NaCl的质量分数由0增加至2%, 4种酚类EDCs的回收率明显增加,而当NaCl的质量分数继续增加至7%时,4种酚类EDCs的回收率略有下降。这是因为将NaCl加入至水样后会形成水合钠离子和水合氯离子,二者削弱了水分子的扩散能力,酚类EDCs很难再与水分子发生接触,从而降低了酚类EDCs在水中的溶解度,使其更容易在吸附材料上富集。因此,实验选择NaCl的质量分数为2%。

### 2.3 方法学验证

#### 2.3.1 基质效应

样品基质与待测目标物共存导致待测目标物在仪器中的响应信号有不同程度增强或抑制的现象叫做基质效应(ME),基质效应会影响分析结果的准确性。为了考察基质效应,将空白水样配制成不同质量浓度(1、5、10、50、100 μg/L)的基质匹配标准溶液,同时用纯水配制相同质量浓度的溶剂混合标准溶液。根据ME=(基质匹配标准曲线斜率/溶剂混合标准曲线斜率-1)×100%评估基质效应^[38]^。当|ME|<20%时,表明存在弱基质效应;当20%≤|ME|≤50%,表明存在中等基质效应;当|ME|>50%时,则表明存在强基质效应。实验结果表明,BPA、4-*t*-OP、NP和4-NP的|ME|值均<20%,为弱基质效应,因此本实验无需采取补偿措施。

#### 2.3.2 线性范围、检出限和定量限

配制4种酚类EDCs质量浓度分别为0.5、1、5、10、50、100 μg/L的模拟水样,考察分析方法性能。以4种酚类EDCs的质量浓度为横坐标(*x*)、峰面积为纵坐标(*y*),建立标准曲线。如表4所示,4种酚类EDCs在0.5~100 μg/L范围内具有良好的线性关系,线性相关系数(*r*^2^)为0.9991~0.9998。以3倍信噪比计算方法的检出限(LOD)、10倍信噪比计算定量限(LOQ), LOD为0.01~0.13 μg/L, LOQ为0.03~0.42 μg/L。

**表 4:** 4种酚类EDCs的线性方程、相关系数、线性范围、检出限和定量限

Analyte	Linear equation	r^2^	Linear range/(μg/L)	LOD/(μg/L)	LOQ/(μg/L)
BPA	y=9755.5x+2848.2	0.9991	0.5-100	0.01	0.03
4-t-OP	y=2120.4x+259.7	0.9998	0.5-100	0.09	0.31
4-NP	y=1473.5x-511.3	0.9996	0.5-100	0.13	0.42
NP	y=10970.0x-2877.1	0.9998	0.5-100	0.04	0.14

*y*: peak area; *x*: mass concentration, μg/L.

#### 2.3.3 回收率和精密度

为了评估分析方法的准确性和可靠性,在空白水样中加入不同体积的4种酚类EDCs混合标准工作液,配制成低、中、高3个加标水平(1、10、50 μg/L)的水样,并进行加标回收试验。在1 d内对3个加标水平样品测定6次,考察日内精密度;连续测定6 d,考察日间精密度。如表5所示,4种酚类EDCs的加标回收率为84.4%~114.2%,日内和日间精密度分别为1.5%~10.6%(*n*=6)和6.1%~13.2%(*n*=6),实验结果说明该分析方法具有较好的准确性和可靠性。

**表 5:** 4种酚类EDCs的加标回收率和日内、日间精密度(*n*=6)

Analyte	Spiked level/ (μg/L)	Recovery/ %	Intra-day RSD/%	Inter-day RSD/%
BPA	1	84.4	8.4	9.9
	10	98.0	10.6	11.9
	50	106.8	3.7	12.6
4-t-OP	1	102.0	9.9	8.2
	10	99.1	1.5	6.1
	50	90.8	4.5	10.3
4-NP	1	90.9	7.2	7.8
	10	97.0	8.3	6.6
	50	97.4	8.3	6.8
NP	1	114.2	3.2	13.2
	10	91.1	8.7	8.0
	50	107.2	3.3	7.0

### 2.4 与文献方法比较

将本文方法与文献报道方法进行比较,结果见表6。相比于基于传统SPE材料的液相色谱-质谱法,本方法所使用的DSPE方法可以使固液分离过程更加简单、快速,同时也可以避免SPE过程中耗时的过柱操作。

**表 6:** 本方法与文献报道方法比较

Analysis method	Sample pretreatment materials	Matrices	Pretreatment time/min	LOD/ (μg/L)	Ref.
SPE-UPLC-MS/MS	amino-functionalized grooved	river and lake water	15.0	0.01	[10]
	polyacrylonitrile nanofiber mat				
	molecular imprinted polymer	serum and urine	>60.0	0.01-0.04	[39]
	Oasis HLB column	wastewater	>50.0	0.002-0.02	[40]
	NAX and C18 mixed packing of column	tap water and effluent water	>200.0	0.00181-0.00332	[41]
	ENVI ChromP column	surface water	>120.0	0.05-0.10	[42]
DSPE-UPLC-MS/MS	UiO-66-C	tap water and surface water	10.5	0.01-0.13	this work

HLB: hydrophile lipophilic balance; NAX: CUNAXOOX; ENVI ChromP: styrene/divinyl benzene copolymer resin.

### 2.5 实际样品分析

为了验证分析方法的实用性,将本方法应用于自来水和地表水的检测。加标水平为1 μg/L的自来水样品经DSPE处理后,4种酚类EDCs均获得了有效富集,DSPE处理前后的色谱图见图5。此外,在自来水样品中未检测到酚类EDCs,而地表水样品中检测到微量的4-NP和NP,检出水平分别为1.38 μg/L和0.26 μg/L,其中NP的检出水平低于美国EPA地表水水质标准限值(6.6 μg/L)。自来水和地表水样品中,4种酚类EDCs在3个加标水平(1、10、50 μg/L)下的回收率分别为77.1%~111.3%和88.2%~116.6%,相对标准偏差分别为2.2%~10.7%和2.2%~14.5%,结果见表7。实验结果表明,所建立的分析方法具有良好的准确度和精密度,能够满足环境水体中酚类EDCs的测定要求。

**图 5:**
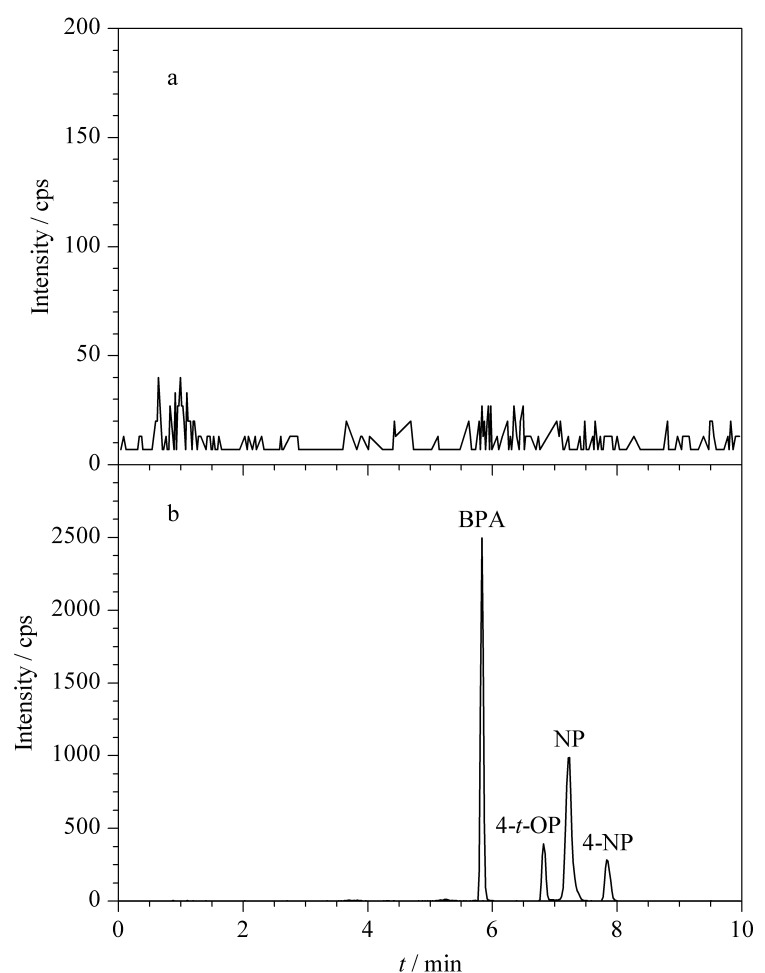
加标水平为1 μg/L的自来水样品经DSPE(a)富集前和(b)富集后的色谱图

**表 7:** 自来水和地表水样品中4种酚类EDCs的分析结果(*n*=3)

Analyte	Spiked level/ (μg/L)	Tap water			Surface water	
		Found/(μg/L)	Recovery (RSD)/%		Found/(μg/L)	Recovery (RSD)/%
BPA	0	ND	/		ND	/
	1	0.85	85.3 (10.7)		0.88	88.2 (7.5)
	10	10.19	101.9 (6.4)		10.29	102.9 (14.5)
	50	53.64	107.3 (5.6)		48.10	96.2 (4.7)
4-t-OP	0	ND	/		ND	/
	1	0.77	77.1 (3.9)		1.07	107.0 (9.1)
	10	7.84	78.4 (5.5)		10.53	105.3 (8.3)
	50	41.92	83.8 (3.5)		53.21	106.4 (5.6)
4-NP	0	ND	/		1.38	/
	1	1.11	111.3 (2.2)		1.11	111.1 (8.0)
	10	9.78	97.8 (4.2)		9.85	98.5 (7.7)
	50	53.42	106.8 (5.0)		56.47	112.9 (8.7)
NP	0	ND	/		0.26	/
	1	1.03	103.6 (9.1)		1.16	116.6 (2.2)
	10	8.59	85.9 (5.3)		9.07	90.7 (9.5)
	50	46.96	93.9 (5.4)		48.25	96.5 (7.3)

ND: not detected; /: no value.

## 3 结论

本研究利用溶剂热法制备了一种MOF衍生多孔碳材料UiO-66-C,并将其作为DSPE吸附剂材料,结合UPLC-MS/MS测定环境水体中的4种酚类EDCs。所建立方法操作简便,萃取速度快,灵敏度高,并能够应用于实际水样的富集分析。研究结果表明,UiO-66-C对酚类EDCs表现出高效的选择吸附性,是一种有前途的萃取吸附材料,为复杂基质中有机污染物的富集浓缩提供了新思路,并扩展了多孔碳材料在环境监测领域中的应用。
